# Novel risk models to predict acute kidney disease and its outcomes in a Chinese hospitalized population with acute kidney injury

**DOI:** 10.1038/s41598-020-72651-x

**Published:** 2020-09-24

**Authors:** Ye-Qing Xiao, Wei Cheng, Xi Wu, Ping Yan, Li-Xin Feng, Ning-Ya Zhang, Xu-Wei Li, Xiang-Jie Duan, Hong-Shen Wang, Jin-Cheng Peng, Qian Liu, Fei Zhao, Ying-Hao Deng, Shi-Kun Yang, Song Feng, Shao-Bin Duan

**Affiliations:** 1 Department of Nephrology, The Second Xiangya Hospital, Central South University; Hunan Key Laboratory of Kidney Disease and Blood Purification, 139 Renmin Road, Changsha, 410011 Hunan China; 2 Information Center, The Second Xiangya Hospital, Central South University, Changsha, 410011 Hunan China; 3 Department of Nephrology, The Third Xiangya Hospital, Central South University, Changsha, 410013 Hunan China; 4 Information Center, The Xiangya Hospital, Central South University, Changsha, 410008 Hunan China

**Keywords:** Kidney diseases, Prognosis, Outcomes research, Risk factors

## Abstract

Acute kidney disease (AKD) is a state between acute kidney injury (AKI) and chronic kidney disease (CKD), but the prognosis of AKD is unclear and there are no risk-prediction tools to identify high-risk patients. 2,556 AKI patients were selected from 277,898 inpatients of three affiliated hospitals of Central South University from January 2015 to December 2015. The primary point was whether AKI patients developed AKD. The endpoint was death or end stage renal disease (ESRD) 90 days after AKI diagnosis. Multivariable Cox regression was used for 90-day mortality and two prediction models were established by using multivariable logistic regression. Our study found that the incidence of AKD was 53.17% (1,359/2,556), while the mortality rate and incidence of ESRD in AKD cohort was 19.13% (260/1,359) and 3.02% (41/1,359), respectively. Furthermore, adjusted hazard ratio of mortality for AKD versus no AKD was 1.980 (95% CI 1.427–2.747). In scoring model 1, age, gender, hepatorenal syndromes, organic kidney diseases, oliguria or anuria, respiratory failure, blood urea nitrogen (BUN) and acute kidney injury stage were independently associated with AKI progression into AKD. In addition, oliguria or anuria, respiratory failure, shock, central nervous system failure, malignancy, RDW-CV ≥ 13.7% were independent risk factors for death or ESRD in AKD patients in scoring model 2 (goodness-of fit, *P*_1_ = 0.930, *P*_2_ = 0.105; AUROC_1_ = 0.879 (95% CI 0.862–0.896), AUROC_2_ = 0.845 (95% CI 0.813–0.877), respectively). Thus, our study demonstrated AKD was independently associated with increased 90-day mortality in hospitalized AKI patients. A new prediction model system was able to predict AKD following AKI and 90-day prognosis of AKD patients to identify high-risk patients.

## Introduction

Acute kidney injury (AKI) is a public health problem which seriously endangers human health^1, 2^. Epidemiological studies showed the incidence rate of chronic kidney disease (CKD) after an episode of AKI was 7.8 events/100 patient-years, and the rate of end stage renal disease (ESRD) was 4.9 events/100 patient-years^3^. The mortality rate of AKI patients was 8.8–23.9%^4–6^. Even with clear definition and staging criteria of AKI (7 days or less)^7^ and CKD (> 90 days)^8^, many patients with renal function and structural changes may not meet the definition. The term acute kidney disease (AKD) has been proposed to define the course of disease after AKI among patients in whom the renal pathophysiologic processes are ongoing. Since the connection between AKI and CKD is well established, the AKD phase represents a time window for potentially initiating key interventions to alter the natural history of kidney disease^9^.

The Kidney Disease: Improving Global Outcomes (KDIGO) guideline advocates follow-up of all patients with AKI^7^. Despite these recommendations, many patients with acute kidney injury have neither received a follow-up assessment, nor received appropriate care^10, 11^, resulting in poor long-term outcomes^12^. However, because not all the AKI patients will progress to ESRD or death^1, 13^, follow-up of all patients hospitalized with AKI could lead to unnecessary use of medical resources.

Compared with AKI patients whose renal function recovers within 7 days, AKD patients suffer from persistent renal impairment and often associate with increased hospital mortality^14^. However, whether the prognosis of AKD patients is different from that of non-AKD patients is poorly understood. We compared the 90-day mortality rates between AKD and non-AKD patients in three affiliated hospitals of Central South University. Then we used population-based routine clinical and laboratory data to derive and validate multivariable prediction models for AKI progression to AKD and prediction of 90-day poor prognosis (ESRD or death) in AKD patients. Our objective was to develop a practical risk stratification approach that can facilitate the clinician's targeted treatment and follow-up to effectively improve the prognosis of AKI patients.

## Methods

### Study design and patient population

**Figure 1:**
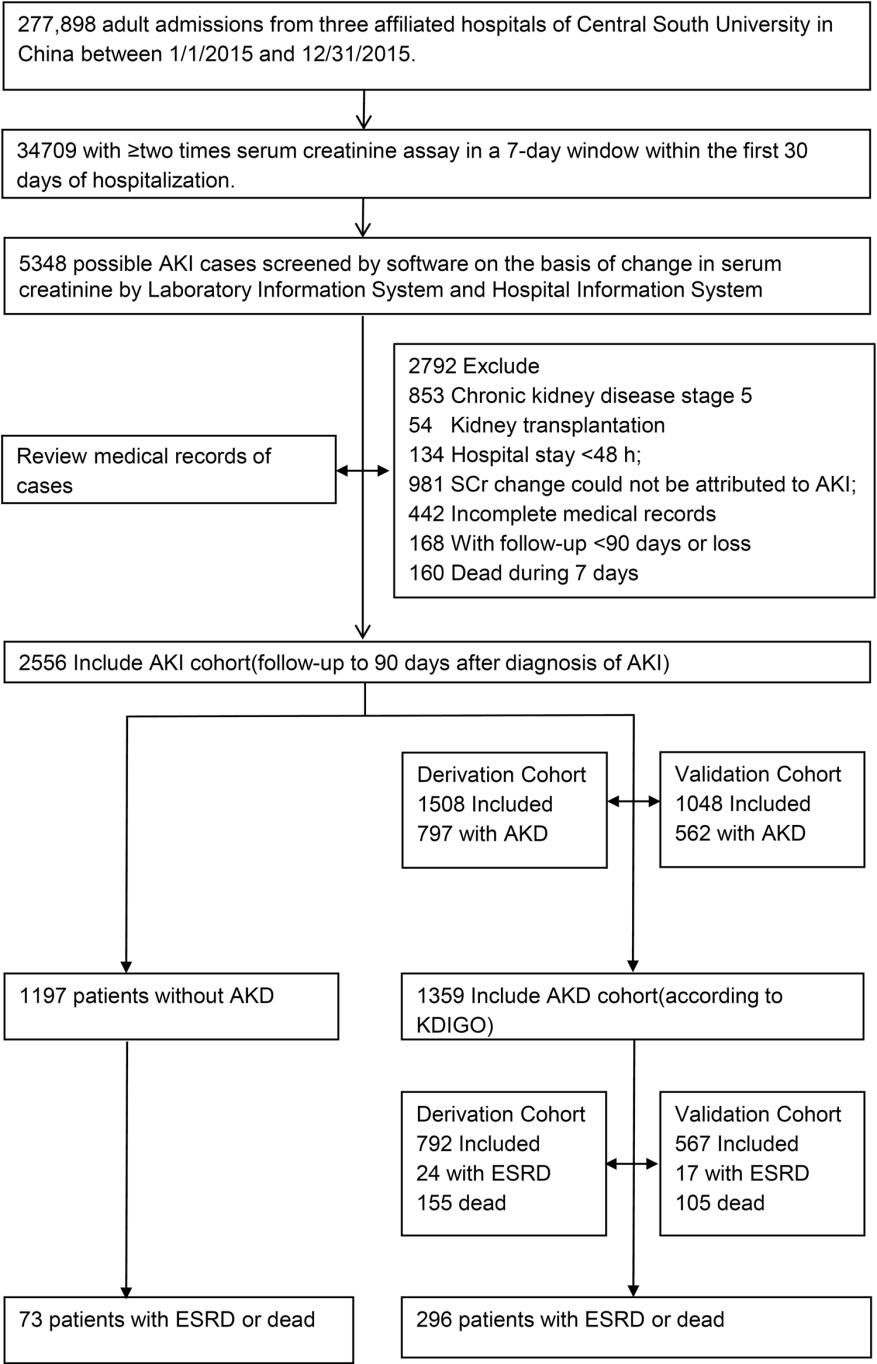
Flow chart of inpatients with AKI progression to AKD and 90-day outcomes.

Our multi-center population-based retrospective study included 277,898 inpatients of three affiliated hospitals of Central South University from January 2015 to December 2015. Among them, there were 103,177 adult patients from the First Xiangya Hospital, 120,090 adult patients from the Second Xiangya Hospital and 54,631 adult patients from the Third Xiangya Hospital, respectively. Figure 1 depicted our flow for selecting study subjects. We selected patients from the cohort who had at least two serum creatinine (SCr) tests within any 7-day window during their first 30 days of hospitalization as the AKI cohort. AKI is defined as an increase in SCr by ≥ 0.3 mg/dl (≥ 26.5 μmol/l) within 48 h; or an increase in SCr to ≥ 1.5 times baseline within the prior 7 days^7^. For patients with multiple hospitalizations, we included only the first hospitalization in the analysis set. All participants were followed up for up to 90-days from admission. The exclusion criteria of participants were: (1) ESRD or requiring renal replacement therapy (dialysis or renal transplantation) before the hospital admission, (2) SCr change not attributed to AKI (e.g., SCr decrease after amputation), (3) hospital stay < 48 h or incomplete medical records, (4) follow-up < 90 days or loss, (5) dead during 7 days. Eventually, 2,556 individuals were selected in our final analyses.

### Definition of primary point and endpoint

The primary point of the observation was 7 days after AKI diagnosis, at which time it was judged whether AKI patients developed non-AKD or AKD. The endpoint was the proportion of death or ESRD with or without long-term renal replacement therapy (dialysis or transplantation) 90 days after AKI diagnosis. These endpoint events were determined by reviewing all relevant medical records (hospital information system, laboratory information system and out-patient records), making phone calls, and sending text messages. All patients were followed up by making phone calls or sending text messages. And 161(6.3%) patients had EHR data during the follow-up period, and we confirmed our follow-up results by reviewing their medical records.

### Definition of data sources

We obtained patient-level data from the electronic hospitalization databases and laboratory databases from the participating hospitals. The hospitalization baseline characteristics consisted of patients’ age, sex, clinical syndromes, causes of AKI, comorbidities**,** laboratory data, AKI stage^7^. The clinical syndromes included oliguria or anuria, hyperkalemia, heart failure, respiratory failure, shock, central nervous system failure, gastrointestinal bleeding^15^. In detail, heart failure included New York Heart Association class I–IV^16^; respiratory failure was defined as hypoxemia with PaO_2_ < 60 mmHg^17^; shock was defined as the systolic arterial pressure less than 90 mmHg or the mean arterial pressure less than 70 mmHg^18^; central nervous system failure referred to encephalopathy with Glasgow coma scale < 13 points without sedation^19^. We divided causes of AKI into hypovolemia^20^, cardio-renal syndromes^21^, hepatorenal syndrome that was defined according to the European Association of Liver Study criteria^22^, sepsis^23^, acute tubular necrosis (ATN), organic kidney disease (except ATN, including acute interstitial nephritis, acute glomerular and vasculitic renal diseases)^24^, and post-renal obstruction. Major comorbidities consisted of hypertension, diabetes and malignancy. The laboratory data included red cell volume distribution width—coefficient of variability (RDW-CV)^25^, platelet (PLT), hemoglobin, blood urea nitrogen (BUN), total bilirubin (TBIL)^26^, albuminuria, the estimated baseline glomerular filtration rate (eGFR) according to modified Modification of Diet in Renal Disease (MDRD) equation^27^. The clinical syndromes, causes of AKI, major comorbidities and AKI stage were evaluated by two trained nephrologists through reviewing medical records and laboratory data and kidney biopsy reports. When discrepancies happened between the two nephrologists, another experienced nephrologist was asked to determine the result. Baseline SCr was defined as the lowest SCr value available between 7 and 365 days prior to admission. For patients who had no reliable SCr record before admission and no evidence of baseline CKD, a back-estimation of the baseline SCr level was performed based on the 4-variable MDRD equation with the assumption of an eGFR of 75 ml/min/1.73 m^2^^28^. To ensure that consistent standards were used during the process, all study investigators received unified training. The information bias was controlled by blind review and evaluation of the medical records.

### Identification and classification of AKD

The included AKI patients were assigned into two cohorts according to outcomes: a non-AKD cohort and an AKD cohort. Non-AKD was defined as a post-AKI SCr within 25% of the baseline (prehospitalization value) and independence from renal replacement therapy^29^. AKD was defined as acute or subacute damage and/or loss of kidney function for a duration of 7–90 days after exposure to an AKI initiating event^9^. AKD cohort included AKD 0C, 1–3 stages. Stage 0C included patients whose SCr levels were 1.25 times higher than baseline but within 1.5 times of baseline levels. AKD 1–3 stages aligned with the 2012 KDIGO AKI staging categories^30^. We used the modified MDRD equation^27^ to describe baseline kidney function in two eGFR subgroups: normal baseline function (eGFRs ≥ 60 ml/min/1.73 m^2^) and decreased baseline kidney function (eGFRs < 60 ml/min/1.73 m^2^).

### Derivation and validation cohorts

Two prediction models were established using multivariable logistic regression, one for inpatients with AKI progression to AKD, the other for predicting the occurrence of ESRD or mortality following AKD patients. In model 1, we randomly selected 60% of the AKI cohort patients as a model derivation cohort and used the remaining 40% as an internal validation cohort. The model 2 was derived from 60% of the AKD cohort and prospectively validated by the remaining 40%.

### Candidate predictor variables

We identified variables for potential inclusion in our models from a review of the literature^14, 24, 31–33^. All the candidate variables in the derivation cohort were included as potential covariates in multivariable logistic regression models. Enter linear regression variable with a significance level at 0.05 for variable retention.

### Model derivation

The corresponding integrals were endowed according to various odds ratio (OR) values of predictor variables. Combined with clinical reality, the scores of each patient were respectively summed up. We then fit a series of reduced models by sequentially removing variables and compared the full multivariable model with the simplified models.

### Prediction model performance

The regression coefficients from the logistic regression models in the derivation sample were fixed. The fitted models were applied to the validation cohort. The Hosmer–Lemeshow test was used for calibration, and discrimination was assessed using the area under the receiver operating characteristic (AUROC). The AUROC analysis was used to calculate cutoff values, sensitivity, and specificity. Finally, the cutoff point was chosen based on the best Youden index which was defined as sensitivity + specificity − 1^34^. According to the cutoff point, whether AKI patients progressing into AKD and 90-day prognosis of AKD patients were predicted; diagnostic efficiency was determined according to the ratio of the predicted outcomes to the actual outcomes.

### Statistical analysis

The collected data were used to establish a qualified database and statistically analyzed by using SPSS 18.0. The data of normal distribution were presented with mean ± standard deviation (mean ± SD). The mean comparison between the two groups was conducted with Student's *t* test. The enumeration data were expressed by rate and analyzed by chi-square test. The rank sum test was used to determine the difference between level data. The independent risk factors that correlated AKI progression to AKD, ESRD or death were analyzed by multivariable logistic regression. Cumulative survival rates between non-AKD and AKD patients were assessed using multivariable Cox regression (forward stepwise selection) after adjusted candidate variables by log-rank test. In addition, the Hosmer–Lemeshow test was used for calibration and discrimination was assessed using AUROC. *P* value less than 0.05 was considered to be statistically significant.

### Ethical considerations

The Medical Ethics Committee of the Second Xiangya Hospital of Central South University approved the study protocol and waived the patient consent. This project has been registered by Chinese Clinical Trial Registry (ChiCTR 1800019857, Registration Date: 12/2/2018). All the methods in the present study were carried out in accordance with guidelines of the Declaration of Helsinki. For this retrospective study, formal informed consent is not required.

## Results

### Cohort description

As shown in Fig. 1, of 277,898 hospitalized patients in three affiliated hospitals of Central South University, 34,709 had at least 2 SCr tests in a 7-day window during their first 30 days of hospitalization. 2,884 had AKI (168 patients lost to follow-up and 160 patients who died within 6 days were included), yielding the incidence of AKI was 8.31% (2,884/34,709) in hospitalized patients. AKI cohort included 308 patients with decreased baseline renal function (eGFR < 60 ml/min/1.73 m2) and 2,248 patients with normal baseline function (eGFR ≥ 60 ml/min/1.73 m^2^). During the 90-day observation period, we found that 1,359 patients progressed into AKD after AKI event, accounting for 3.92% (1,359/34,709) of hospitalized patients and 53.17% (1,359/2,556) of the AKI patients, respectively. In AKD cohort, incidence of ESRD was 3.02% (41/1,359) and the mortality rate was 19.13% (260/1,359).

### Characteristics of patients

Baseline characteristics of the derivation and validation cohorts in AKI and AKD are described in Tables 1 and 2. Data were stratified by baseline kidney function for those with or without AKD. Additional data for patient characteristics in eGFR subgroups are provided in Supplementary Table S1 online.

**Table 1:** Baseline characteristics of the derivation and validation cohorts of AKI patients.

Characteristics	Cohort, No. (%) of AKI patients	
	Derivation (*n* = 1508)	Validation (*n* = 1,048)
**Demographics**		
Age, mean (SD), years	53.7 (16.6)	55.0 (16.2)
≥ 65 years, No. (%)	419 (27.8)	324 (30.9)
Gender (women), No. (%)	556 (36.9)	397 (37.9)
**Clinical syndromes**		
Oliguria or anuria^a^, No. (%)	320 (21.2)	231 (22)
Hyperkalemia^b^, No. (%)	145 (9.6)	101 (9.6)
Heart failure^c^, No. (%)	257 (17)	195 (18.6)
Respiratory failure^d^, No. (%)	208 (13.8)	140 (13.4)
Shock^e^, No. (%)	236 (15.6)	174 (16.6)
Central nervous system failure^f^, No. (%)	264 (17.5)	189 (18)
Gastrointestinal bleeding^g^, No. (%)	95 (6.3)	63 (6)
**Causes of AKI, No. (%)**		
Hypovolemia	768 (50.9)	542 (51.7)
Cardio-renal syndromes	38 (2.5)	15 (1.4)
Hepatorenal syndrome	33 (2.2)	24 (2.3)
Sepsis	152 (10.1)	96 (9.2)
Organic kidney disease (except ATN)	219 (14.5)	153 (14.6)
Acute tubular necrosis	124 (8.2)	81 (7.7)
Post-renal obstruction	80 (5.3)	65 (6.2)
Multi-factorial	95 (6.3)	72 (6.9)
**Laboratory data**^h^		
RDW-CV ≥ 13.7%	777 (51.5)	742 (70.8)
PLT < 100 or > 300 × 10^9^/l	563 (37.3)	365 (34.8)
Hemoglobin < 90 g/l	401 (26.6)	286 (27.3)
ALB < 30 g/l	529 (35.1)	364 (34.7)
BUN ≥ 7.14 mmol/l	1,056 (70.0)	742 (70.8)
The baseline eGFR^i^ < 60 ml/min/1.73 m^2^	200 (13.3)	108 (10.3)
**Albuminuria**^**g**^**, No. (%)**		
Normal	1,122 (74.4)	767 (73.2)
Mild	320 (21.2)	244 (23.3)
Heavy	66 (4.4)	37 (3.5)
**Acute kidney injury stage**^**k**^**, No. (%)**		
Stage 1	596 (39.5)	420 (40.1)
Stage 2	368 (24.4)	247 (23.6)
Stage 3	544 (36.1)	381 (36.4)

^a^Oliguria or anuria (Urine volume < 400 or 100 ml/24 h).

^b^Hyperkalemia (Serum K + peak value > 5.5 mmol/l).

^c^Heart failure (defined as New York Heart Association class I–IV).

^d^Respiratory failure (hypoxemia with PaO_2_ < 60 mmHg)

^e^Shock (the systolic arterial pressure is less than 90 mmHg or the mean arterial pressure is less than 70 mmHg).

^f^Central nervous system failure (encephalopathy with Glasgow coma scale < 13 points without sedation.).

^g^Gastrointestinal bleeding (upper gastrointestinal bleeding and lower gastrointestinal bleeding).

^h^The worst value was taken within 7 days.

^**i**^ The estimated GFR according to modified glomerular filtration rate estimating equation.

^**j**^Normal albuminuria is defined by an albumin: dipstick urinalysis protein negative (−); mild, dipstick urinalysis protein trace of 1+ or 2+; and heavy, dipstick urinalysis protein of 3+ or higher.

^k^According to three categories of KDIGO staging system based on the highest SCr value identified during hospitalization.

**Table 2:** Baseline characteristics of the derivation and validation cohorts of AKD patients.

Characteristic	Cohort, No. (%) of AKD Patients	
	Derivation (*n* = 792)	Validation (*n* = 567)
**Demographics**		
Age, mean (SD), years	55.4 (16.7)	55.4 (17.4)
Age ≥ 65 years, No. (%)	257 (32.4)	193 (34)
Gender (women), No. (%)	256 (32.3)	198 (34.9)
**Clinical data**		
Oliguria or anuria^a^, No. (%)	293 (37)	201 (35.4)
Hyperkalemia^b^, No. (%)	94 (11.9)	91 (16.0)
Heart failure^c^, No. (%)	160 (20.2)	132 (23.3)
Respiratory failure^d^, No. (%)	153 (19.3)	107 (18.9)
Shock^e^, No. (%)	159 (20.1)	118 (20.8)
Central nervous system failure^f^, No. (%)	168 (21.2)	117 (20.6)
Gastrointestinal bleeding^g^, No. (%)	67 (8.5)	39 (6.9)
**Cause of AKI, No. (%)**		
Hypovolemia	279 (35.2)	211 (37.2)
Cardio-renal syndromes	22 (2.8)	13 (2.3)
Hepatorenal syndrome	29 (3.7)	18 (3.2)
Sepsis	105 (13.3)	69 (12.2)
Organic kidney disease (except ATN)	187 (23.6)	132 (23.3)
Acute tubular necrosis	70 (8.8)	59 (10.4)
Post-renal obstruction	61 (7.7)	41 (7.2)
Multi-factorial	39 (4.9)	25 (4.4)
**Comorbidities, No. (%)**		
Hypertension	248 (31.3)	178 (31.4)
Diabetes	169 (21.3)	107 (18.9)
Malignancy	135 (17.0)	112 (19.8)
**Laboratory data**^b^		
RDW-CV ≥ 13.7%	420 (53)	301 (53.1)
BUN ≥ 7.14 mmol/l	691 (87.2)	468 (82.5)
The baseline eGFR^i^ < 60 ml/min/1.73 m^2^	90 (11.4)	60 (10.6)
TBIL(μmol/l)		
0 < 20	583 (73.6)	421 (74.3)
20–32	72 (9.1)	52 (9.2)
33–101	66 (8.3)	53 (9.3)
102–204	28 (3.5)	17 (3.0)
> 204	43 (5.4)	24 (4.2)
Albuminuria^j^, No. (%)		
Normal	513 (64.8)	382 (67.4)
Mild	228 (28.8)	145 (25.6)
Heavy	51 (6.4)	40 (7.1)
**Acute kidney injury stage**^**k**^**, No. (%)**		
Stage 1	125 (15.8)	92 (16.2)
Stage 2	179 (22.6)	135 (23.8)
Stage 3	488 (61.6)	240 (60.0)

^a^Oliguria or anuria (urine volume < 400 or 100 ml/24 h).

^b^Hyperkalemia (Serum K + peak value > 5.5 mmol/l).

^c^Heart failure (defined as New York Heart Association class I–IV).

^d^Respiratory failure (hypoxemia with PaO_2_ < 60 mmHg).

^e^Shock (the systolic arterial pressure less than 90 mmHg or the mean arterial pressure less than 70 mmHg).

^f^Central nervous system failure (encephalopathy with Glasgow coma scale < 13 points without sedation).

^g^Gastrointestinal bleeding (upper gastrointestinal bleeding and lower gastrointestinal bleeding).

^h^The worst value was taken within 7 days.

^**i**^The estimated GFR according to modified glomerular filtration rate estimating equation.

^**j**^ Normal albuminuria is defined by an albumin: dipstick urinalysis protein negative (−); mild, dipstick urinalysis protein trace of 1+ or 2+; and heavy, dipstick urinalysis protein of 3+ or higher.

^k^According to three categories of KDIGO staging system based on the highest SCr value identified during hospitalization.

### Survival analysis

**Figure 2:**
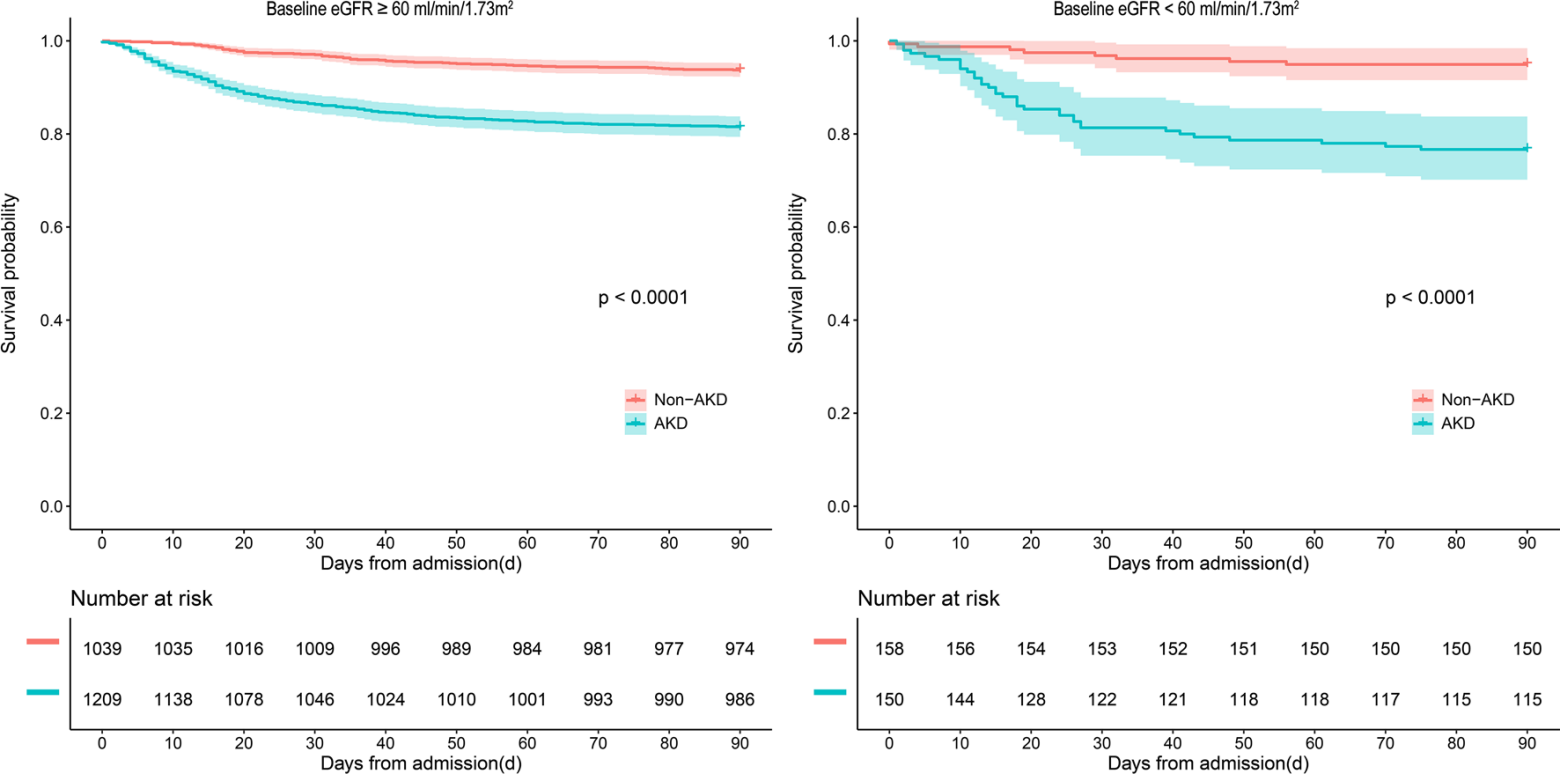
Cumulative survival of non-AKD and AKD cohort stratified by baseline kidney function.

Adjusted 90-day hazard ratio (HR) of mortality for AKD versus no AKD was 1.980 (95% CI 1.427–2.747). Figure 2 presented the comparison of crude mortality based on baseline kidney function between AKD and no AKD patients. Regardless of baseline kidney function, 90-day mortality was higher for AKD patients versus no AKD. In those with normal baseline kidney function, the independent risk factors of 90-day mortality were age, oliguria or anuria, central nervous system failure, cardio-renal syndromes, malignancy, RDW-CV ≥ 13.7%, TBIL (20–32 μmol/l, > 204 μmol/l), AKD, respiratory failure, shock. However, in those with decreased baseline kidney function, only respiratory failure, shock, heart failure and hemoglobin were the independent risk factors of 90-day mortality; additional data for mortality of eGFR subgroups are available in Supplementary Table S2 online.

### Prediction of AKD in the AKI derivation cohort (model 1)

By using multivariable logistic regression analysis in bootstrapped samples of the AKI derivation cohort, eight variables were found to be associate with a higher risk of progression to AKD, including age, gender, hepatorenal syndrome, organic kidney disease, oliguria or anuria, respiratory failure, BUN, acute kidney injury stage. The corresponding integrals of OR values of the independent risk factors for AKI progression to AKD were endowed after adjusted by cutoff point and each patient’s score was calculated according to the formula (Table 3).

**Table 3:** Eight-variable risk index for AKD following hospitalization with AKI in the AKI derivation cohort.

Predictors	Odds ratio (95% CI)	P value	Classification	Points
Age	1.758 (1.286–2.404)	< 0.001	< 65	0
			≥ 65	2
Gender	0.545 (0.405–0.732)	< 0.001	Woman	0
			Man	1
Hepatorenal syndrome	8.205 (2.710–24.842)	< 0.001	No	0
			Yes	7
Organic kidney disease			No	0
	2.175 (1.260–3.754)	0.005	ATN	2
	6.262 (3.934–9.967)	< 0.001	Except ATN	6
Oliguria or Anuria	3.104 (1.997–4.826)	< 0.001	No	0
			Yes	3
Respiratory failure	1.976 (1.281–3.049)	0.002	No	0
			Yes	2
BUN	1.933 (1.414–2.642)	< 0.001	< 7.14	0
			≥ 7.14	2
Acute kidney injury stage	1	–	Stage 1	0
	3.289 (2.399–4.508)	< 0.001	Stage 2	3
	18.787 (12.813–27.546)	< 0.001	Stage 3	7
Maximum points				32

The formula of Model 1 is as follow: Score of AKI to AKD = the points of age + the points of gender + the points of hepatorenal syndrome + the points of organic kidney disease + the points of oliguria or anuria + the points of respiratory failure + the points of BUN + the points of acute kidney injury stage. The scoring criteria are as follow: 2 points for age ≥ 65 years old; 1 points for gender(man); 7 points for hepatorenal syndrome; 2 points for ATN and 6 points for organic kidney disease(except ATN); 3 points for oliguria or anuria; 2 points for respiratory failure; 2 points for BUN ≥ 7.14 mmol/l; 3 points for Acute kidney injury stage2 and 7 points for Acute kidney injury stage3.The total scores of each patient were calculated.

### Prediction model 1 performance in the AKI validation cohort

As shown in Table 4, the model 1 remained well calibrated in the AKI derivation cohort and had the overall goodness-of-fit (*P* = 0.930). The discrimination results demonstrated that model 1 showed a large AUROC (0.879 ± 0.009, 0.879 ± 0.011) and had stable sensitivity (81%, 82%) and specificity (81%, 80%) in AKI derivation cohort and AKI validation cohort, respectively. We defined the cutoff point according to the best Youden index (0.62). If scores were greater than the cutoff point (7), the AKI patients were judged to develop into AKD. The derivation cohort and validation cohort were divided into two risk stratifications, respectively, according to the cutoff point: 0–7 (low-risk patients) and > 7 (high-risk patients). The diagnostic efficiency showed that the predictability of the scoring system was reliable (both 81% in derivation cohort and validation cohort).

**Table 4:** Predictive performance of prediction model system for inpatients with AKI progression to AKD and 90-day outcomes in the derivation and validation cohorts.

	Goodness- of-fit (P value)	AUROC ± SD (95% CI)	Youden index	Cut off point	Sensitivity (%)	Specificity (%)	Diagnostic efficiency (%)
**Model 1**							
Derivation (N = 1,508)	3.075 (0.930)	0.879 ± 0.009 (0.862–0.896)	0.62	7	81	81	81
Validation (N = 1,048)	–	0.879 ± 0.011 (0.858–0.900)	0.62	7	82	80	81
**Model 2**							
Derivation (N = 792)	11.862 (0.105)	0.845 ± 0.017 (0.813–0.877)	0.55	4	79	76	76
Validation (N = 567)	–	0.809 ± 0.024 (0.763–0.856)	0.52	4	78	74	75

### Prediction of mortality or ESRD in the AKD derivation cohort (model 2)

The further multivariable logistic regression analysis indicated that oliguria or anuria, respiratory failure, shock, central nervous system failure, malignancy, RDW-CV ≥ 13.7% were the independent risk factors of ESRD or death in bootstrapped samples of the AKD derivation cohort. The corresponding integrals of various OR values of the independent risk factors for ESRD or death in patients with AKD were endowed and each patient’s score was calculated according to the formula (Table 5).

**Table 5:** Six-variable risk index for 90-day mortality or ESRD outcomes in patients with AKD.

Predictors	Odds ratio (95%CI)	P value	Classification	Points
Oliguria or anuria	2.075 (1.374–3.133)	0.001	No	0
			Yes	2
Respiratory failure	5.194 (3.206–8.413)	< 0.001	No	0
			Yes	5
Shock	2.593 (1.578–4.260)	< 0.001	No	0
			Yes	3
Central nervous system failure	2.091 (1.273–3.432)	0.004	No	0
			Yes	2
Malignancy	2.86 (1.743–4.693)	< 0.001	No	0
			Yes	3
RDW-CV (%)	2.492 (1.623–3.827)	< 0.001	< 13.7	0
			≥ 13.7	2
Maximum points				17

The formula of Model 2 is as follow: Score of AKI to AKD = the points of oliguria or anuria + the points of respiratory failure + the points of shock + the points of central nervous system failure + the points of malignancy + the points of RDW-CV. The scoring criteria are as follow: 2 points for oliguria or anuria; 5 points for respiratory failure; 3 points for shock; 2 points for Central nervous system failure, 3 points for malignancy and 2 points for RDW-CV ≥ 13.7%. The total scores of each patient were calculated.

### Prediction model 2 performance in the AKD validation cohort

The model 2 remained well calibrated in the AKI derivation cohort and had the overall goodness-of-fit (0.105). The cutoff point was defined according to the best Youden index (0.55). If scores were greater than the cutoff point (4), the AKD patients were judged to develop into ESRD or death. It was predicted that the AUROC of 90-day mortality and ESRD in AKD derivation and validation cohorts, were 0.845 (95% CI 0.813–0.877) and 0.809 (95% CI 0.763–0.856), respectively. They also had higher stable sensitivity (79%; 78%), specificity (76%; 74%) and diagnostic efficiency (76%; 75%), showing that the predictability of the scoring system was reliable (Table 4).

## Discussion

We found that all-cause 90-day mortality rate of AKD patients markedly increased, compared with that of non-AKD patients. We identified risk factors of AKI progression to AKD and AKD progression to ESRD or death. Based on dynamic evolution of AKI, we established two models to form an early warning system to predict AKD following hospitalization with AKI and 90-day prognosis of AKD patients.

AKI is associated with an increased risk of CKD and ESRD, and elevated long-term risk of mortality^35, 36^. Our results showed the overall incidence of AKI in hospitalized patients was 8.31% (2,884/34,709), and this data was consistent with previous reports of 2.4–8.1% in all adult inpatients^5^, which indicated that the further studies based on AKI cohort was credible. AKD is conceptualized as post-AKI to represent an important transition period after AKI^9^. As the link between AKI and CKD is firmly established, the AKD period represents the time window wherein critical interventions might be initiated to alter the natural history of kidney disease. Up to date, there are few studies focusing on AKD. Scarce studies have been made on the risk factors and prognosis for AKD. Sawhney et al.^32^ found that 43.2% of AKI patients (1,330/3,081) completely recovered within 7 days; however, during the 90-day follow-up period after AKI, 20.4% of patients (629/3,081) partly recovered; 49.1% of patients (1513/3,081) showed no significant change in Scr; 9.9% of patients (305/3,081) deteriorated and 20.5% of patients (632/3,081) died. It indicates that a considerable part of patients will develop AKD after AKI and then enter CKD. Fujii et al. found that nearly 1% of hospitalized patients developed subacute kidney injury (s-AKI, equivalent to AKD) and that s-AKI was independently associated with increased hospital mortality, but patients with s-AKI had a better outcome and were less likely to require renal replacement therapy than AKI patients^14^. However, another study showed development of AKD was associated with higher mortality and needed for renal replacement therapy in cardiac surgery patients^37^. In this study, we found that 3.92% of hospitalized patients developed AKD and the incidence of AKD after AKI was 53.17%, which were higher than previous report in Japan^14^. We further discovered that 90-day all-cause mortality rate was significantly higher in AKD patients (vs non-AKD patients) irrespective of baseline kidney function. Besides, although more patients with AKI eventually developed into ESRD, ESRD was a rare outcome in the absence of AKD. All the results indicated that AKD was a critical predictor of adverse outcomes after AKI. We can explore more risk factors and intervention links that affect prognosis in this time window to facilitate recovery and minimize continuing damage. Therefore, it is necessary for clinician to identify the independent risk factors for AKI progression to AKD and predict the adverse prognosis of AKD patients.

In this study, based on AKD definition, we first established one model for prediction of AKI progression to AKD in AKI cohort and then established the other model to predict 90-day adverse outcomes (ESRD or death) in patients with AKD. An 8-variable model and corresponding risk index (variables including age, gender, hepatorenal syndrome, organic kidney disease, oliguria or anuria, respiratory failure, BUN, and acute kidney injury stage) showed the reliable performance for predicting whether inpatients with AKI would develop AKD later in derivation and validation cohorts. What is more, AKD patients who had oliguria or anuria, respiratory failure, shock, central nervous system failure, malignancy, RDW-CV ≥ 13.7% would probably progress to ESRD or death in the short term. These two models performed well in both derivation and the validation cohorts with relatively large AUROC, stable sensitivity and specificity, as well as high diagnostic efficiency. Models are typically considered useful for clinical decision-making when the AUROC is higher than 0.70, and AUROC of these two models both exceeds 0.80, suggesting these models could support clinical decision-making. In addition, we tried to improve the accuracy of the models based on fewer variables alone.

This analysis was consistent with and further extended previous work. Mizuguchi et al.^37^ showed that SCr-based AKI stages could identify high-risk patients of AKI progression to AKD after cardiac surgery and the patients with higher AKI stages were more likely to develop AKD in a graded manner. This study only focused on AKI stages in cardiac surgery patients, but did not conduct multivariable analysis on different etiologies of AKI. James et al.^31^ have established and validated prediction models for progression of AKI to advanced CKD. Tangri et al. and Drawz et al.^38, 39^ have developed models to predict ESRD in patients with CKD. Models for prognostic stratification and risk adjustment for predicting mortality after AKI have been reported^40^. Demirjian et al. and Poukkanen et al.^41, 42^ provided assessment scores to predict 60-day mortality or 1-year mortality in critically ill patients. Our previous study also established a scoring model for predicting 90-day prognosis in patients with AKI^24^. However, these studies failed to dynamically predict the prognosis of AKI patients in different stages. In addition, these studies also did not consider the possibility that the sustained renal injury status after AKI might worsen the prognosis of patients. To our knowledge, we are the first investigators to describe the major adverse kidney outcomes (90-days mortality or ESRD) of AKD following hospitalization with AKI, and to dynamically observe the prognosis of AKI patients in different stages. Our study also innovatively compared the differences in 90-day all-cause mortality between AKD and non-AKD patients under different baseline kidney function, which indicated a key intervention point after AKI events to promote recovery and reduce sustained renal damage might occur in AKD.

A strength of this study was that incidence and major adverse kidney outcomes of AKD patients were revealed, which clarified the scientific significance of AKD. Based on a large, population-representative AKI cohort, we further established an early warning system (two models) to predict incidence of AKD and 90-day outcomes. The predictor variables used in these two models and risk indices may be readily ascertained at the time of hospital discharge, making it possible to identify high-risk patients in time. What is more, these two risk-prediction models provide theoretical basis for treatment of AKI patients in different stages. Clinicians can identify high-risk patients for targeted follow-up in the community which can avoid the waste of medical resources. The model 1 can identify high-risk patients of AKD in AKI patients so that clinicians can timely intervene, correct reversible risk factors and communicate with patients to plan for medical treatment; the model 2 can screen high-risk patients with AKD for predicting 90-day mortality or ESRD rate. It is helpful to guide short-term prognostic assessment and follow-up, especially during transition to outpatient medical care.

Nevertheless, our study has several limitations. First, like most retrospective studies on AKI, our study did not use urine output to identify AKI because urinary data were not available for most patient. Second, inpatients were all adults without children. Third, the models established in our study are static models, which can be further informed by dynamic models of time-varying data, particularly for 90-day outcomes. Fourth, recall bias existed in this retrospective study. Because all patients were followed up by making phone calls or sending text messages. And 161(6.3%) patients had EHR data during the follow-up period, and we confirmed our follow-up results by reviewing their medical records. Finally, models were derived and validated in cohorts from three hospitals of Central South University as a retrospective multi-center study, and lack of generalizability to patients in other regions. Therefore, multi-center prospective trials are still necessary to evaluate the accuracy of these two models in predicting AKI to AKD and 90-day outcomes of AKD patients.

In conclusion, we discovered that AKD was independently associated with increased 90-day mortality in hospitalized AKI patients. More importantly, an early warning system for prognosis of inpatients with AKI using routine laboratory data was able to predict AKD following hospitalization with AKI and 90-day prognosis of AKD patients to identify high-risk patients. These risk prediction models provide an accurate but simple strategy that could be used to stratify patients into clinically meaningful risk groups at the time of hospital discharge and guide further management in the community^43^. The utility of these models in clinical care requires further research.

## Supplementary information

Supplementary file1

## Data Availability

The datasets generated during and/or analyzed during the current study are available from the corresponding author on reasonable request.
