# Supplementary material for: Novel risk models to predict acute kidney disease and its outcomes in a Chinese hospitalized population with acute kidney injury
Source: Sci Rep. 2020 Sep 24;10:15636. doi: 10.1038/s41598-020-72651-x (PMC7519048; doi:10.1038/s41598-020-72651-x)
Supplement: Supplementary file 1 — Supplementary file1 [file 41598_2020_72651_MOESM1_ESM.pdf]

Article title:

Novel risk models to predict acute kidney disease and its outcomes in a Chinese hospitalized population with acute kidney injury

Author names:

Ye-Qing Xiao, MS; Wei Cheng, MD; Xi Wu, MS; Ping Yan, MD; Li-Xin Feng, MD; Ning-Ya Zhang, MS; Xu-Wei Li, MS; Xiang-Jie Duan, MS; Hong-Shen Wang, MD; Jin-Cheng Peng, MS; Qian Liu, MD; Fei Zhao, MD; Ying-Hao Deng, MD; Shi-Kun Yang, MD; Song Feng , MS; Shao-Bin Duan, MD.

Supplementary Table S1

Characteristics by baseline kidney function in the non-AKD and AKD group

Characteristic	Of 2,556 Patients in the Entire AKI Cohort by Baseline eGFR					
	Baseline Decreased Kidney Function: eGFR < 60 mL/min/1.73 m2			Normal Baseline Kidney Function: eGFR≥60 mL/min/1.73 m2		
	No-AKD group (*n*=158)	AKD group (*n*=150)	*P* value	No-AKD group (*n*=1039)	AKD group (*n*=1209)	*P* value
**Demographics**						
Age, mean±SD, years	53.02 ±15.62	55.02 ±17.12	0.001	52.84±16.49	58.08 ±15.73	0.828
Gender (Women), No. (%)	405（39.0%）	37（30.9%）	<0.001	94（59.5%）	81（54.0%）	<0.331
**Clinical syndromes , No. (%)**						
Oliguria or anuria [a]	54（5.2%）	44（37.0%）	<0.001	3（1.9%）	47（31.3%）	<0.001
Hyperkalemia [b]	53（5.1%）	16（13.4%）	<0.001	8（5.1%）	2（15.3%）	0.003
Heart failure [c]	140（13.5%）	25（21.3%）	<0.001	20（12.7%）	35（23.3%）	0.014
Respiratory failure [d]	72（6.9%）	224（18.5%）	<0.001	16（10.1%）	3（24.0%）	0.001
Shock [e]	119（11.5%）	234（19.4%）	<0.001	14（8.9%）	43（28.7%）	<0.001
Central nervous system failure [f]	144（13.9%）	245（63.0%）	<0.001	24（15.2%）	40（26.7%）	0.013
Gastrointestinal bleeding [g]	45（4.3%）	92（7.6%）	0.001	7（4.4%）	14（9.3%）	0.008
**Causes of AKI, No. (%)**						
Hypovolemia	697（67.1%）	43（36.1%）	<0.001	123（77.8%）	53（35.3%）	<0.001
Cardio-renal syndromes	17（1.6%）	33（2.7%）	0.080	1（0.6%）	2（1.3%）	0.532
Hepatorenal syndrome	10（1.0%）	41（3.4%）	<0.001	0（0.0%）	6（4.0%）	0.011
Sepsis	66（6.4%）	15（12.7%）	<0.001	8（5.1%）	20（6.5%）	0.012

**Organic kidney disease (except ATN)**	47（4.5%）	27（23.1%）	<0.001	6（3.8%）	40（26.7%）	<0.001
**Acute tubular necrosis**	70（6.7%）	110（9.1%）	0.040	6（3.8%）	19（12.7%）	0.004
**Post-renal obstruction**	37（3.6%）	92（7.6%）	<0.001	6（3.8%）	10（6.7%）	0.257
**Multi-factorial**	95（9.1%）	82（6.8%）	0.038	8（5.1%）	3（2.0%）	0.148
**Comorbidities, No. (%)**						
**Hypertension**	277（26.7%）	375（31.0%）	0.023	43（27.2%）	51（34.0%）	0.196
**Diabetes**	189（18.2%）	236（19.5%）	0.422	20（12.7%）	40（26.7%）	0.002
**Malignancy**	260（25.0%）	207（44.3%）	<0.001	62（39.2%）	40（26.7%）	0.009
**Laboratory data [h]**						
**RDW,（%）**	14.45±2.98	14.68±5.01	0.177	14.66±3.68	15.388±7.00	0.285
**PLT，10^9/l**	186.64±110.43	171.62±108.76	0.750	184.77±115.97	173.566±104.33	0.484
**Hemoglobin，mean±SD, g/l**	111.19±27.74	107.88±28.09	0.410	108.46±25.809	97.227±26.35	0.827
**ALB, mean±SD, g/l**	34.179±8.75	31.40±11.18	0.543	34.61±7.04	31.406±7.75	0.102
**BUN, mean±SD, mmol/L**	9.44±7.33	17.23±11.99	<0.001	9.42±5.83	15.86±9.04	<0.001
**TBIL, mean±SD, μmol/L**	30.659±59.80	39.67±99.28	<0.001	27.23±46.58	42.13±94.66	<0.001
**Albuminuria [i], No. (%)**						<0.001
**Normal**	866（83.3%）	783（64.8%）	/	128（81.0%）	112（74.7%）	/
**Mild**	164（15.8%）	336（27.8%）	/	27（17.1%）	37（24.7%）	/
**Heavy**	9（0.9%）	90（7.4%）	/	3（1.9%）	1（0.7%）	/
**Acute kidney injury stage [j], No. (%)**			<0.001			<0.001
**Stage 1**	700（67.4%）	186（15.4%）	/	100（63.3%）	30（20.0%）	/
**Stage 2**	258（24.8%）	272（22.5%）	/	43（27.2%）	42（28.0%）	/
**Stage 3**	81（7.8%）	750（62.1%）	/	15（9.5%）	78（52.0%）	/
**Length of hospital stay, median, d**	18.63±17.39	19.41±18.48	0.016	23.677±15.84	21.08±18.19	0.813

[a] Oliguria or anuria (Urine volume <400 or 100 mL/24 h), [b] hyperkalemia (Serum K+ peak value > 5.5 mmol/L), [c] heart failure (defined as New York Heart Association class I-IV), [d] respiratory failure (hypoxemia with PaO2< 60 mm Hg), [e] shock (the systolic arterial pressure is less than 90 mm Hg or the mean arterial pressure is less than 70 mm Hg), [f] central nervous system failure (encephalopathy with Glasgow coma scale < 13 points without sedation.),

[g] gastrointestinal bleeding (upper gastrointestinal bleeding and lower gastrointestinal bleeding).

[h] The worst value was taken within 7 days.

[i] Normal albuminuria is defined by an albumin: dipstick urinalysis protein negative(-); mild, dipstick urinalysis protein trace of 1+ or 2+; and heavy, dipstick urinalysis protein of 3+ or higher.

[j] According to three categories of KDIGO staging system based on the highest SCr value identified during hospitalization.

Supplementary Table S2

Independent predictor of mortality: multivariate cox regression analysis using forward stepwise selection

Characteristic	Of 2556 Patients in the Entire AKI Cohort by Baseline eGFR			
	Normal Baseline Kidney Function: eGFR≥60 mL/min/1.73 m^2		Baseline Decreased Kidney Function: eGFR＜60 mL/min/1.73 m^2	
	HR (95%CI)	P value	HR (95%CI)	P value
Age	1.389(1.089-1.771)	0.008	/	/
Oliguria or Anuria	1.376(1.057-1.789)	0.017	/	/
Central nervous system failure	2.566(1.916-3.437)	<0.001	/	/
Cardio-renal syndromes	1.979(1.136-3.445)	0.016	/	/
Malignancy	1.906(1.460-2.488)	<0.001	/	/
RDW-CV	1.324(1.026-1.708)	0.031	/	/
TBIL (20-32 umol/L)	1.562(1.093-2.232)	0.014	/	/
TBIL (＞204umol/L)	2.744(1.826-4.122)	<0.001	/	/
AKD	1.941(1.436-2.623)	<0.001	/	/
Respiratory failure	3.612(2.683-4.864)	<0.001	4.153(1.960-8.797)	0.000
Shock	1.603(1.186-2.166)	0.002	2.787(1.285-6.047)	0.009
Heart failure	/	/	2.027(1.072-3.831)	0.030
Hemoglobin	/	/	2.006(1.055-3.815)	0.034
